# Positive outcomes with neoadjuvant chemotherapy in the management of colovesical fistula in cancer: a case report and literature review

**DOI:** 10.3389/fphar.2023.1284316

**Published:** 2024-01-15

**Authors:** Song Tang, Xinjun Li, Donghua Guo, Fan Zhuo

**Affiliations:** ^1^ Department of Gastrointestinal Surgery, The First Affiliated Hospital of Guangdong Pharmaceutical University, Guangzhou, Guangdong, China; ^2^ Department of Oncological Surgery, Guangzhou Royallee Cancer Center, Guangzhou, Guangdong, China; ^3^ Medical Imaging Unit, Guangzhou Royallee Cancer Center, Guangzhou, Guangdong, China

**Keywords:** cancerous colovesical fistula, sigmoid colon cancer, neoadjuvant chemotherapy, treatment, case report

## Abstract

Colovesical fistula (CVF) is usually developed from colonic diverticulitis, followed by tumor. Traditional surgery is usually completed in one or more stages. For complex cancerous CVF, radical resection is more difficult. We report a 62-year-old male patient diagnosed with sigmoid colon cancer combined with sigmoid vesical fistula. In the course of treatment, in addition to conventional surgery, neoadjuvant chemotherapy (NAC) was innovatively used. The sigmoid tumor and fistula were significantly shrunken. Radical surgery achieved negative margins.

## Introduction

About two-thirds of CVFs develop from colonic diverticulitis, and other causes include colorectal tumors, bladder tumors, Crohn’s disease, radiation therapy, and trauma (Zizzo et al., 2022). Fistulas occur most often between the sigmoid colon and the bladder (Steier et al., 1973; Larsen et al., 1996; Kobayashi et al., 2003). The initial symptoms of CVF are usually related to the urinary tract, such as pneumaturia, fecaluria, and urinary tract infection symptoms (Steier et al., 1973; Zizzo et al., 2022).

In patients with operable CVF, surgery is traditionally performed in one, two, or three stages (Steele et al., 1979; Ashrafi and Sotelo, 2019; Zizzo et al., 2022; Xu et al., 2023). In 1973, Steier et al. (Steier et al., 1973) proposed that it was safer to complete the operation in three stages, that is, proximal colostomy was performed first, followed by colectomy, fistula resection and bladder repair, and finally colostomy closure. However, for tumor patients, the occurrence of CVF means that it is already in the local advanced stage, and radical resection is difficult. Preoperative neoadjuvant therapy (Either folinic acid, fluorouracil and oxaliplatin (FOLFOX) or capecitabine and oxaliplatin (XELOX)) for colon cancer has achieved positive results in a number of studies, and has been proven to reduce tumor stage and increase R0 removal rate (Foxtrot Collaborative, 2012; Gosavi et al., 2021; Hindson, 2023; Morton et al., 2023).

In the case we now report, FOLFOX-based NAC was innovatively used in addition to proximal colostomy prior to radical surgery. Sigmoid tumor and fistula were significantly shrunken, which reduced the difficulty of surgery. Negative incisal margin was obtained during radical surgery.

### Case presentation

A 62-year-old male patient came to our hospital for frequent urination, urgent urination, odynuria and fecaluria for 4 months. A computed tomography (CT) performed at a local hospital 1 month ago indicated CVF, and the etiology was considered to be sigmoid or bladder cancer. After anti-infection treatment, the symptoms of urinary tract infection are slightly relieved, but there is still fecaluria. After admission to our hospital, the abdominal physical examination showed that the abdomen was soft, no tenderness and rebound pain, and no abdominal mass was palpable. He weighs 54 kg and is 160 cm tall.

**FIGURE 1:**
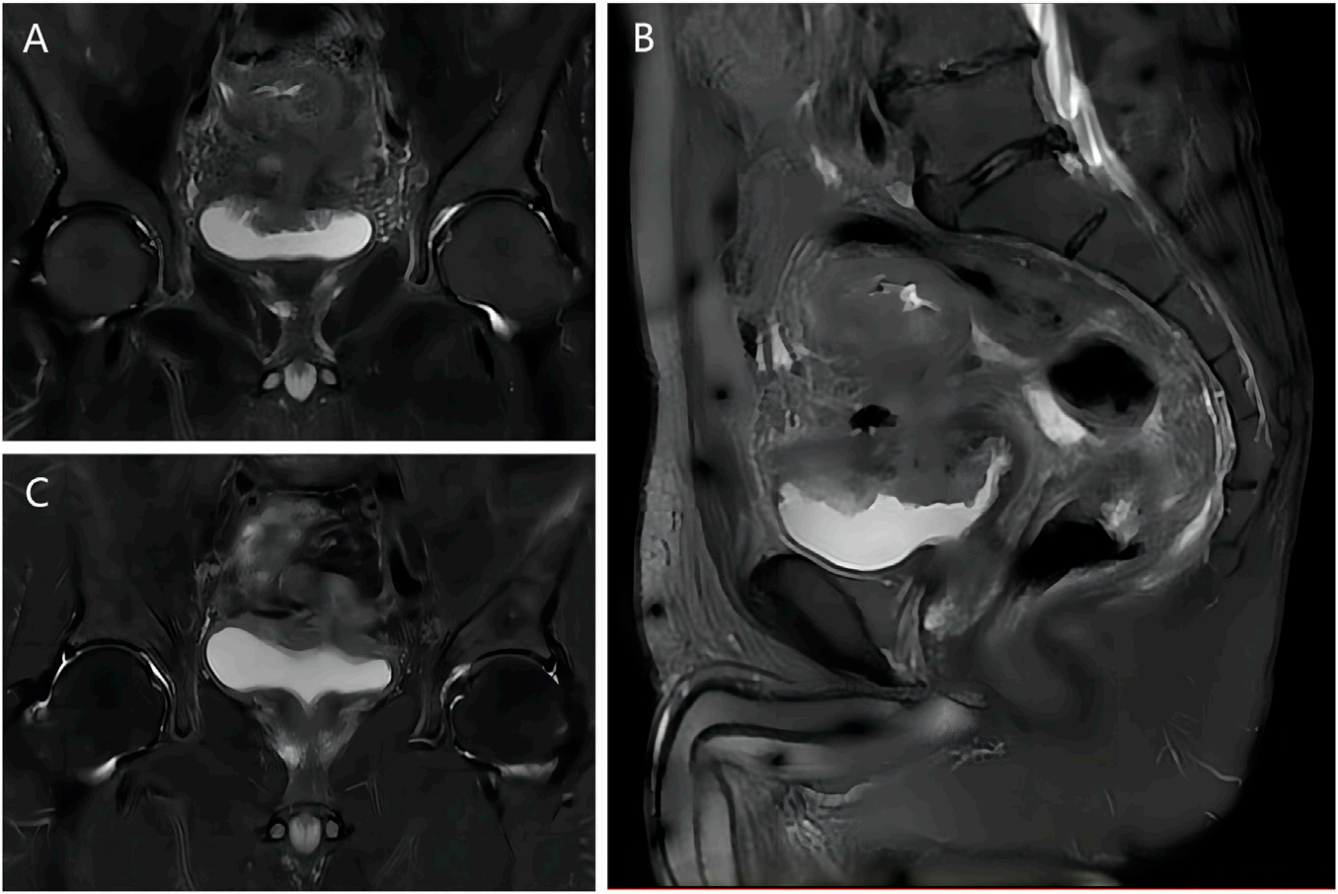
MRI images of patient before and after NAC. **(A)** T2 coronal image before NAC; **(B)** T2 sagittal image before NAC; **(C)** T2 coronal image after NAC.

Urine analysis showed increased white blood cell and bacterial counts, and carcinoembryonic antigen and carbohydrate antigen 199 were in the normal range. Pelvic magnetic resonance imaging (MRI) (Figures 1A, B) showed that the sigmoid mass was about 86 mm in length and the tube wall was thickened, about 21 mm at the thickest point, which was consistent with colon cancer. Involving the top of the bladder and fistula formation; Perisigmoid, bilateral iliac vessels, and retroperitoneal lymph nodes were shown, and partial metastasis was considered. Computed tomography urography (CTU) showed that the excretion of bilateral ureters and bilateral renal pelvis calyces was normal. The bladder wall is not uniformly thickened with gas buildup. There were no hepatopulmonary metastases on chest CT and abdominal CT.

During colonoscopy, a nodular raised lesion was observed at a distance of 20 cm from the anus in the sigmoid colon. The surface appeared congested and swollen in a nodular pattern, with growth encircling the intestinal wall, leading to significant luminal narrowing. The endoscope could not be advanced due to this. Biopsy samples were obtained and sent for pathology, which indicated a high-grade adenomatous intraepithelial neoplasia with localized carcinoma transformation. However, due to the superficial sampling, the depth of infiltration cannot be determined.

We invited experts in medical oncology, minimally invasive intervention, imaging and surgical anesthesiology to hold a multidisciplinary consultation. The diagnosis of sigmoid cancer complicated with sigmoid vesical fistula was basically clear. The pathological stage was T4bN0M0. Considering the large size of sigmoid tumor and serious adhesion between tumor and bladder, it was decided to complete the operation in three stages and give 4 cycles of NAC before radical surgery. The regimen was mFOLFOX6 (oxaliplatin 85mg/m2, calcium folinate 400mg/m2, fluorouracil 400mg/m2 bolus, then fluorouracil 2400mg/m2 by continuous infusion). If the tumor recedes, then radical surgery is followed by 8 cycles of adjuvant chemotherapy with the same regimen.

We first performed laparoscopic descending colostomy (hartman). Then, 4 cycles of NAC were given without serious chemotherapy side effects. Pelvic MRI (Figure 1C) showed significant shrinks of sigmoid mass and fistula (about 60 mm in length and 18 mm at the thickest part of the intestinal wall). At the same time, the symptoms of urinary tract infection were significantly relieved, and fecaluria disappeared.

One month after the end of NAC, the patient returned to the hospital for laparoscopic radical resection of sigmoid carcinoma, partial resection and repair of bladder, closure of descending colostomy, and ileostomy. Surgery went well. The bleeding was only about 100 mL.

**FIGURE 2:**
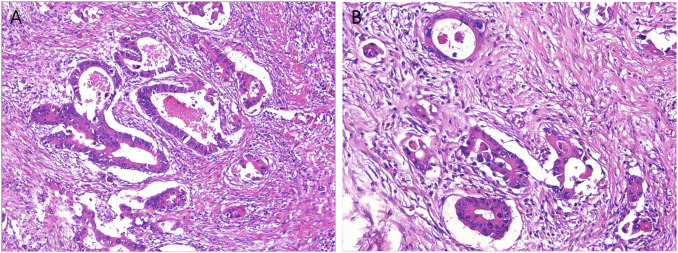
Pathological results of the patients after radical surgery. **(A)** The structure of the glandular ducts was slightly different from that of normal glands, showing moderate differentiation; **(B)** Lympho-vascular invasion is visible.

Pathological results of radical surgery (Figure 2): differentiated adenocarcinoma of sigmoid, positive lympho-vascular invasion, positive nerve invasion, negative proximal incisal margin, negative distal incisal margin, negative periannular (radial) mesangial incisal margin, lymph node metastasis (2/27), adenocarcinoma invasion of bladder mucosa and base.

Immunohistochemical results (see Table 1 for the complete spelling of abbreviations): ① Sigmoid tissue: MLH-1 (+), PMS-2 (+), MSH-2 (+), MSH-6 (+), Braf V600E (−), P53 (mutant), PD-L1 (22C3) (CPS<1), KI-67 (+, 80%); ② Bladder tissue: P40 (−), CK7 (partial +), CK20 (+), GATA3 (−), P53 (mutant), KI-67 (+, 90%), CDX2 (+).

**TABLE 1:** List of abbreviations for immunohistochemistry.

Abbreviations	Complete spelling
MLH-1	MutL Homolog 1
PMS-2	Post- meiotic segregation increased 2
MSH-2	MutS homolog 2
MSH-6	MutS homolog 6
CPS	Combined Positive Score
CK	Cytokeratin
GATA3	GATA binding protein 3
CDX2	Caudal type homeobox transcription factor 2

The recovery after radical surgery was smooth, no symptoms of fecaluria and urinary tract infection occurred, urine analysis of bacteria and white blood cell counts returned to the normal range, the surgical wound healed well, up to now, no anastomotic leakage. The patient was very satisfied with the results of the treatment. 8 cycles of adjuvant chemotherapy with the mFOLFOX6 regimen will be followed, and ileostomy closure will be performed within 1 month after completion of chemotherapy.

### Discussion

CVFs often develop from colonic diverticulitis, followed by tumors (Zizzo et al., 2022). Fistulas mostly form between the sigmoid colon and the bladder (Steier et al., 1973; Larsen et al., 1996; Kobayashi et al., 2003). Due to anatomical differences, CVFs are more common in males, while in females, the occurrence of CVFs is often associated with cervical cancer (Chen et al., 2010). Our patient has a CVF caused by adenocarcinoma of the sigmoid colon.

CVF can be initially diagnosed based on the history, and its clinical symptoms begin in the urinary tract rather than the colon, and are most often characterized by pneumosuria, fecaluria, and urinary tract infection (Steier et al., 1973; Zizzo et al., 2022). Fecaluria is a characteristic symptom of CVF, and pneumouria can also be seen in emphysematous cystitis (Thomas et al., 2007). In addition, CVF can be diagnosed by CT, MRI, cystoscopy, colonoscopy, barium enema, cystography, and poppy seed test (Jarrett and Vaughan, 1995; Kwon et al., 2008; Tang et al., 2012; Zizzo et al., 2022). In our case, both CT and MRI showed obvious fistulas. In MRI, the most common form is the presence of an abscess between the intestinal wall and the bladder wall, the second form is the presence of a visible trace between the affected colon and the bladder, and the third form is the complete disappearance of the fat layer between the affected colon and the bladder wall (Tang et al., 2012). As can be seen from Figure 1, our case belongs to the second type.

CVFs can be treated conservatively and surgically, depending on the patient’s physical condition, lesion site, and complications (Zizzo et al., 2022). For benign CVFs without sepsis and without symptoms, especially in Crohn’s disease, conservative treatment may be an option (Scozzari et al., 2010; Golabek et al., 2013; Cochetti et al., 2018; Zizzo et al., 2022). The vast majority of patients are treated by surgery, and open, laparoscopic and robotic surgery are the options, while the effect of endoscopic treatment remains to be evaluated (Zizzo et al., 2022). The main purpose of surgery is to remove the diseased intestinal segment and repair the fistula, which can be completed in one or multiple stages (Scozzari et al., 2010; Golabek et al., 2013; Cochetti et al., 2018). In most cases, one-stage surgery is preferred, which can improve patients’ quality of life (Cochetti et al., 2018; Zizzo et al., 2022). For patients with pelvic abscesses, advanced malignancies, or prior radiotherapy, multistage surgery may be an option (Steier et al., 1973; Steele et al., 1979; Cochetti et al., 2018; Ashrafi and Sotelo, 2019; Zizzo et al., 2022; Xu et al., 2023). Our case is sigmoid colon cancer, so we chose to complete the surgery in three stages.

To date, we have seen only one report of NAC and targeted therapy for CVF, which used FOLFOX plus panitumumab and resulted in significant reduction of the primary tumor, but no description of fistula effect (Tomizawa et al., 2017). We believe that neoadjuvant chemotherapy is less common in previous reports, probably because colvesical fistula is more common in colonic diverticulitis, which does not require perioperative chemotherapy. However, for patients with less common colon tumors, the presence of a CVF indicates an advanced local tumor stage. If the colon tumor is large and firmly adheres to the bladder, achieving a negative surgical margin after curative resection becomes challenging.

Despite concerns from a study about the potential for overtreatment and delaying subsequent tumor management in patients (Tsang and Lau, 2022), multiple investigations have already demonstrated the feasibility and safety of NAC in patients with locally advanced colon cancer (Foxtrot Collaborative, 2012; Gosavi et al., 2021; Hindson, 2023; Morton et al., 2023). NAC induces tumor regression, downstages the tumor stage, enhances the R0 resection rate, and allows assessment of the tumor’s response to chemotherapy, thereby informing postoperative adjuvant chemotherapy strategies (Foxtrot Collaborative, 2012; Gosavi et al., 2021; Hindson, 2023; Morton et al., 2023). In our case, there was significant tumor regression (>30%) after, and according to the Response Evaluation Criteria in Solid Tumors (RECIST) (Therasse et al., 2000; Litière et al., 2017), the sigmoid tumor achieved clinical partial response. Negative margins were obtained during radical resection. Thus, the feasibility of NAC in the treatment of carcinoma CVF was proved.

Although the patient in this case has achieved better results, there are still some deficiencies. First, because CT and pelvic MRI had confirmed the diagnosis of CVF, and the patient also had symptoms of urinary tract infection, no cystoscopy was performed during the diagnosis process. The colonoscopy also failed to enter because of the narrowing of the colon. Therefore, we did not obtain visual images of the fistula before surgery. Second, this is a retrospective study, and the feasibility and safety of NAC in patients with cancerous CVF need to be further explored in large-scale, multi-center prospective studies.

## Conclusion

For CVF caused by colon cancer, NAC may be used before radical surgery if the tumor is large and the bladder is severely attached, but the feasibility and safety need to be further explored by large-scale, multi-center prospective studies.

## Data Availability

The original contributions presented in the study are included in the article/Supplementary materials, further inquiries can be directed to the corresponding author.
